# Endogenous Spatial Attention Modulates the Magnitude of the Colavita Visual Dominance Effect

**DOI:** 10.1177/20416695211027186

**Published:** 2021-07-12

**Authors:** Aijun Wang, Heng Zhou, Yuanyuan Hu, Qiong Wu, Tianyang Zhang, Xiaoyu Tang, Ming Zhang

**Affiliations:** Department of Psychology, 12582 Soochow University, Suzhou, China; Research Center for Psychology and Behavioral Sciences, Soochow University, Suzhou, China; Department of Psychology, 66339 Suzhou University of Science and Technology, Suzhou, China; School of Public Health, Medical College of 12582 Soochow University, Suzhou, China; School of Psychology, 66523 Liaoning Normal University, Liaoning Collaborative Innovation Center of Children and Adolescents Healthy Personality Assessment and Cultivation, 66523 Liaoning Normal University, Dalian, China; Department of Psychology, 12582 Soochow University, Suzhou, China; Research Center for Psychology and Behavioral Sciences, Soochow University, Suzhou, China

**Keywords:** attention, endogenous/exogenous, multisensory/cross-modal processing, spatial cognition, visuo-auditory interactions

## Abstract

The Colavita effect refers to the phenomenon wherein people tend to not respond to an auditory stimulus when a visual stimulus is simultaneously presented. Although previous studies have shown that endogenous modality attention influences the Colavita effect, whether the Colavita effect is influenced by endogenous spatial attention remains unknown. In the present study, we established endogenous spatial cues to investigate whether the size of the Colavita effect changes under visual or auditory cues. We measured three indexes to investigate the effect of endogenous spatial attention on the size of the Colavita effect. These three indexes were developed based on the following observations in bimodal trials: (a) The proportion of the “only vision” response was significantly higher than that of the “only audition” response; (b) the proportion of the “vision precedes audition” response was significantly higher than that of the “audition precedes vision” response; and (c) the reaction time difference of the “vision precedes audition” response was significantly higher than that of the “audition precedes vision” response. Our results showed that the Colavita effect was always influenced by endogenous spatial attention and that its size was larger at the cued location than at the uncued location; the cue modality (visual vs. auditory) had no effect on the size of the Colavita effect. Taken together, the present results shed light on how endogenous spatial attention affects the Colavita effect.

Although streams of information from multiple sensory modalities often reach us simultaneously, our brain does not give equal weight to different sensory modalities. Visual information more frequently receives preferential processing than other sensory information and eventually dominates awareness and behavior (de Gelder & Bertelson, 2004; Schmid et al., 2011). One intriguing example of vision’s dominance over audition is the Colavita effect (Colavita, 1974). In the classic paradigm of the Colavita effect, visual, auditory, and audiovisual stimuli are randomly presented, and participants are instructed to press one key for the visual stimulus, another for the auditory stimulus, and both for the audiovisual stimulus. Although participants could respond accurately to the unimodal visual and auditory stimuli, they often failed to respond to the auditory component of an audiovisual stimulus and responded almost exclusively to the visual component. In Colavita (1974), some of the participants reported after the experiment that they did not even perceive the auditory component in the bimodal trials.

The Colavita effect has been revealed to be robust irrespective of a variety of experimental manipulations, such as stimulus intensity, stimulus type, stimulus position, response demands, and arousal (Colavita, 1974; Colavita et al., 1976; Colavita & Weisberg, 1979; Koppen et al., 2008; Koppen & Spence, 2007a; Sinnett et al., 2007). However, it has been shown that the manipulation of endogenous attention can modulate the size of the Colavita effect (Colavita, 1974; Sinnett et al., 2007; Spence, 2009). Researchers have found that although attentional manipulation can reduce or even eliminate the Colavita effect, it cannot reverse the visual dominance effect (Koppen & Spence, 2007c, 2007d; Sinnett et al., 2007). However, in previous studies, researchers have focused on endogenous modality attention, manipulating attention via instructions or goals through the use of the endogenous attentional selectivity mechanism to allocate to a specific modality in multisensory streams (Tang et al., 2016).

Paying attention to a specific modality can accelerate information processing in low-level cortical areas; this effect is referred to as the prior-entry effect (Vibell et al., 2007). Some studies have found that behavioral and event-related potential responses to audiovisual stimuli increased when participants paid attention to visual (Wu et al., 2009), auditory (Li et al., 2010), or audiovisual streams (Giard & Peronnet, 1999). Other studies have demonstrated that the effect of multisensory integration on behavioral performance can be attenuated or even eliminated under conditions of modality-specific selective attention (Mozolic et al., 2008; Wu et al., 2012). In a study of sensory dominance in multisensory integration, it was noted that the Colavita effect can be affected by endogenous modality attention (Sinnett et al., 2007), specifically, when endogenous attention was biased to the visual modality, the size of the Colavita effect became larger, while when it was biased to the auditory modality, the size of the Colavita effect became smaller. Recently, a study showed that endogenous attention toward one specific sensory modality modulates both the direction and size of the Colavita effect (Fang et al., 2020). However, Talsma (2015) noted that attention can influence multisensory processing not only by modality attention but also by spatial attention. Attention was directed to one particular location or multiple specific locations by guidance or visual central cues; the two types of attention were referred to as selective spatial attention and divided spatial attention. Interestingly, endogenous spatial attention can promote the response to location targets in both selective and divided conditions (Li et al., 2015, Wu et al., 2009).

Our study aimed to investigate whether the Colavita effect is affected by endogenous spatial attention. Previous studies have shown that endogenous spatial attention influences multisensory integration, specifically, the redundancy effect, in which responses to the simultaneous presentation of stimuli from multiple sensory systems can be faster and more accurate than responses to the same stimuli presented in isolation (Kinchla, 1974; Li et al., 2010, 2015; Wu et al., 2009). However, multisensory integration includes not only the redundancy effect of multimodality information integration but also the modality dominance of multimodality information competition (Colavita, 1974). The Colavita effect is one of the visual dominance effects of multisensory competition; it is the phenomenon wherein our brain, which is being inundated concurrently by streams of information from multiple sensory modalities, does not give equal weight to different modalities (Huang et al., 2015). This effect is proposed to arise from asymmetric facilitation and inhibition between modalities (Hirst et al., 2018). As a typical paradigm of multisensory integration, whether and how the Colavita effect is affected by endogenous spatial attention still remains unknown.

In the present study, we manipulated a cue, the direction of an arrow, to induce endogenous spatial attention. Talsma et al. (2010) proposed that top-down directed attention can influence multisensory processing (Alsius et al., 2005, 2007; Harrar et al., 2014; Talsma et al., 2007, 2010; Talsma & Woldorff, 2005). Therefore, based on task instructions or informative central visual cues, attention can be focused on a spatial location, such as the left or right side of fixation, which is called selective spatial attention. Previous studies have reported that this endogenous attentional selectivity can facilitate responses to unimodal (visual, V, or auditory, A) signals at the attended (expected) spatial locations relative to the unattended (unexpected) locations (Coull & Nobre, 1998; Li et al., 2012; Posner, 1980; Tang et al., 2013). This analogous attention effect has also been found for stimuli from multiple sensory modalities, for example, the simultaneous presentation of auditory and visual stimuli (audiovisual, AV; Li et al., 2010; Wu et al., 2009). In the present study, the cue-target paradigm was used to explore the effect of endogenous spatial attention on the Colavita effect. We established endogenous visual (a central arrow pointing left or right in Experiment 1) spatial cue to explore how endogenous spatial attention influence the Colavita effect. However, since the Colavita effect is a multisensory integration effect generated by the competition between visual and auditory information, visual cues may interfere with the Colavita effect. Therefore, we conducted Experiment 2 using an auditory (the semantically meaningful sounds “right” or “left”) spatial cue to exclude the influence of cue modalities on the Colavita effect. Although we used both visual and auditory cues, they worked the same way, directing participants’ attention to specific locations; therefore, we assumed that the Colavita effect was greater for the cued location than for the uncued location and unrelated to the modality of the cue.

In our study, we used three indexes to measure the size of the Colavita effect. The first is the classic index used in previous studies to measure the size of the visual dominance effect: the difference between the proportion of Visual_Only (where only the “visual key” is pressed) errors and the proportion of Auditory_Only (where only the “auditory key” is pressed) errors in bimodal trials. However, visual dominance can also appear in the proportion of first response in two keys (e.g., Koppen & Spence, 2007a, 2007b, 2007c, 2007d; Van Damme et al., 2009), which we used as our second index. In a large proportion of bimodal trials, which have been excluded from classic analyses of patterns of error, participants press the two response keys corresponding to both the visual and auditory components. In most of these bimodal trials, although participants make both responses, they cannot press the visual and auditory keys strictly simultaneously: Either the visual response precedes the auditory response or vice versa. Thus, by post hoc categorization of bimodal trials into a “vision precedes audition” response and an “audition precedes vision” response and by comparing the proportions of the two types of responses, we obtain a new valid indictor of the visual dominance effect (Fang et al., 2020; Huang et al., 2015; Li et al., 2017; Yue et al., 2015). The third index is the time difference between the two reactions. The difference in reaction time (RT) between pressing the visual key first and the auditory key second has been found to be significantly larger than the difference in the response time between pressing the auditory key first and the visual key second, demonstrating the visual dominance effect (Huang et al., 2015).

## Experiment 1

### Method

#### Participants

We recruited 33 healthy volunteers (21 female, 12 male, aged 18–26 years) in the study. They were all right handed, with normal hearing and normal or corrected-to-normal visual acuity. None of them had a history of neurological or psychiatric disorders. All participants gave informed consent prior to the experiment in accordance with the Declaration of Helsinki and were paid afterward. This study was approved by the Ethics Committee of Department of Psychology, Soochow University.

To test the suitability of the sample size, we performed a sensitivity analysis of the two-tailed paired sample *t* test in G*power 3.1.9.2 (Faul et al., 2007, 2009). Input parameters: ɑ err prob = 0.05, power (1–β err prob) = 0.80, and total sample size = 33. Output parameter: Cohen’s *d* = 0.50. The results showed that we have enough power to achieve moderate effect, so our sample size was appropriate.

#### Apparatus and Materials

The visual cue was a white central arrow pointed at the left or right (0.7°×1.5° of visual angle) which was presented for 300 ms, and then the central fixation point appears in the center of the screen for a duration of 50 ms. The visual target was a black sphere (2.98°×2.98° of visual angle) which was presented for 50 ms, both them were presented on a 17-inch CRT monitor controlled by Presentation software (Neurobehavioral Systems Inc., Albany, CA; https://www.neurobs.com/). The auditory target was a pure tone (4000 Hz, 55–68 dB, measured via a sound level meter) which was presented for 50 ms via two headsets. Amplitude enveloping was applied to the first and last 5 ms of the pure tone, using the Cool Edit software. Responses were collected with a keyboard.

#### Procedure

**Figure 1.**
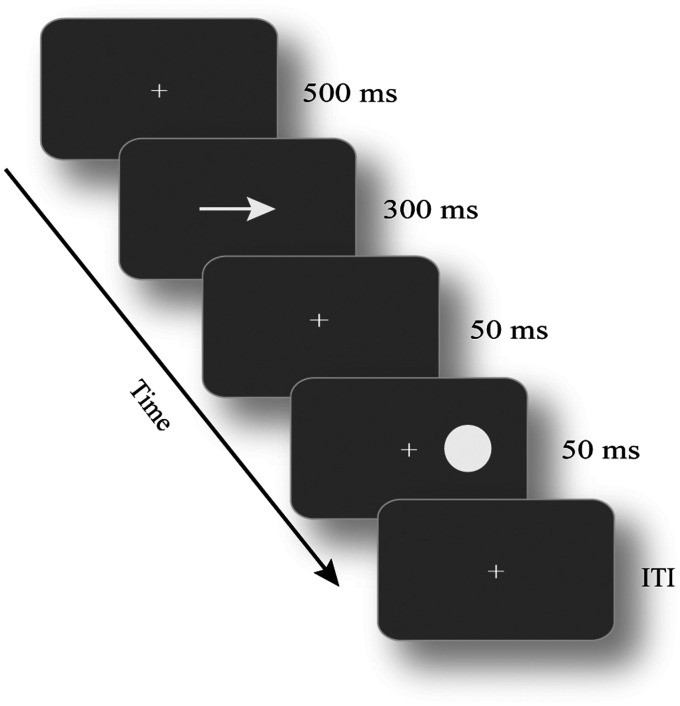
The stimulus and experimental procedure in Experiment 1. The visual cue was an arrow pointing to the left or right. There were three types of target stimulus: visual stimuli, auditory stimuli, and bimodal stimuli; ITI was randomized from 1,400 ms to 1,900 ms. ITI = intertrial interval.

Participants sat 60 cm away from the monitor in a dimly lit room (see Figure 1). They were instructed to put their chin on a chin rest and maintain central fixation throughout the experiment. Before the target was presented, the visual cue was presented. The cue pointed to the left or right locations, followed by the target presented at the cued or uncued location. There were three types of targets: (a) unimodal visual stimuli, in which participants were instructed to press “F” key once they saw the visual target, (b) unimodal auditory stimuli, in which participants were instructed to press “J” key once they heard the auditory target, and (c) bimodal audiovisual stimuli, in which participants were instructed to press “F” key and “J” key at the same time as possible. The mapping between the auditory/visual stimulus and the two response keys was counterbalanced across participants. There were 2,100 trials in total, among which the proportion of visual, auditory, and audiovisual stimuli was 2:2:1 (i.e., 40% visual stimuli, 40% auditory stimuli, and 20% bimodal stimuli). The three types of trials were randomly presented. The validity of the cue was 80%, that is, the cue and the target appeared the same location. The intertrial interval was randomized from 1,400 ms to 1,900 ms (i.e., 1,400, 1,650, and 1,900 ms). Participants practiced for 5 minutes before the formal experiment; the formal experiment lasted 120 minutes.

#### Data Analyses

We used methods that have been used in recent years to analyze the Colavita effect (Fang et al., 2020; Huang et al., 2015; Li et al., 2017; Yue et al., 2015). In addition to the two types of unimodal trials, the bimodal trials were categorized into the following six types according to participants’ responses: (a) the Visual_Auditory trials, in which participants first responded to the visual component and then to the auditory component; (b) the Auditory_Visual trials, in which participants first responded to the auditory component and then to the visual component; (c) the Visual_Only trials, in which participants responded to the visual component only; (d) the Auditory_Only trials, in which participants responded to the auditory component only; (e) the “Simultaneous” trials, in which participants responded simultaneously to the auditory and the visual components (the visual and auditory responses were made within 5 ms); and (f) the “Missed” trials, in which no responses were recorded. The proportion of each type of bimodal trial was calculated as the proportion between the number of each type of bimodal trial and the overall number of bimodal trials.

Although most previous studies have used the difference in proportion between Visual_Only trials (where only the “F” key is pressed) and Auditory_Only trials (where only the “J” key was pressed) in bimodal trials to measure the size of the Colavita effect (e.g., Koppen & Spence, 2007a, 2007b, 2007c, 2007d; Van Damme et al., 2009), the Colavita effect can also appear in RT data (see Egeth & Sager, 1977; Koppen & Spence, 2007c). For instance, in most bimodal trials, although participants made both responses, they could not press the visual and auditory keys absolutely simultaneously: They could exhibit either the “vision precedes audition” response or the “audition precedes vision” response. Thus, by comparing the proportions of the Visual_Auditory trials and Auditory_Visual trials, the Colavita effect can be measured. More interestingly, previous studies have found that the RT difference in pressing the visual key before the auditory key is greater than the RT difference in pressing the auditory key before the visual key (Huang et al., 2015). The formulas used to calculate the RT difference are as follows: In Visual_Auditory trials, ΔRT_1_ = RT_(auditory response)_ – RT_(visual response)_; in Auditory_Visual trials, ΔRT_2_ = RT_(visual response)_ – RT_(auditory response)_, ΔRT_1_ > ΔRT_2_. By comparing ΔRT between Visual_Auditory trials and Auditory_Visual trials, the Colavita effect can be measured.

Therefore, we can investigate the influence of endogenous spatial attention on the Colavita effect through three indexes. In this study, first, we analyzed the proportions of trials when the participants pressed only a single key and conducted 2 (cue validity: cued vs. uncued) × 2 (type of incorrect bimodal trial: Visual_Only vs. Auditory_Only) repeated-measures analysis of variance (ANOVA). We verified the existence of the Colavita effect by observing whether the main effect type of incorrect bimodal trial was significant. Then, we used the difference in proportion between the Visual_Only trials and the Auditory_Only trials as the size of the Colavita effect to conduct a comparison between the cued and uncued locations. Second, we analyzed the proportions of trials in which the participants pressed two keys at different times and conducted a 2 (cue validity: cued vs. uncued) × 2 (type of correctly responded bimodal trial: Visual_Auditory vs. Auditory_Visual) repeated-measures ANOVA. We verified the existence of the Colavita effect by observing whether the main effect of type of correctly responded bimodal trial was significant. Then, we used the difference in proportion between the Visual_Auditory trials and Auditory_Visual trials as the size of the Colavita effect to conduct a comparison between the cued and uncued locations. For analysis of RT, the trials with omissions, incorrect responses and RTs exceeding 3 *SD*s from the mean RT for each condition were excluded from further analysis. We used simple effect analysis with Bonferroni correction to further analyze the interaction effect; we report the effect size by using $\eta_{\text{p}}^{2}$ for ANOVA and Cohen’s *d* for *t* test to ensure statistical validity.

### Results

#### Proportions of Different Types of Bimodal Trials

**Figure 2.**
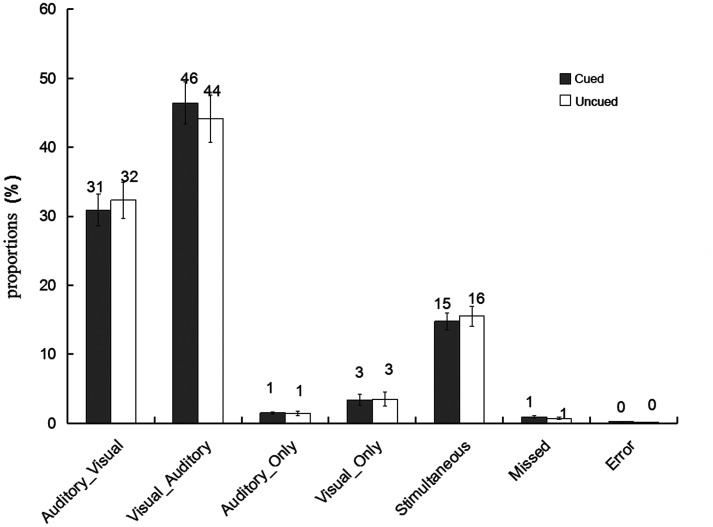
The proportions of the six different types of conditions in the bimodal trials at the cued and the uncued location. Visual_Auditory trials, in which visual responses preceded auditory responses; Auditory_Visual trials, in which auditory responses preceded visual responses; Visual_Only trials, in which only visual responses were made; Auditory_Only trials, in which only auditory responses were made; Simultaneous trials, in which participants responded simultaneously to the auditory and the visual components (RT difference less than 5 ms); and Missed trials, in which no responses were recorded (the error bars indicate SEs).

**Figure 3.**
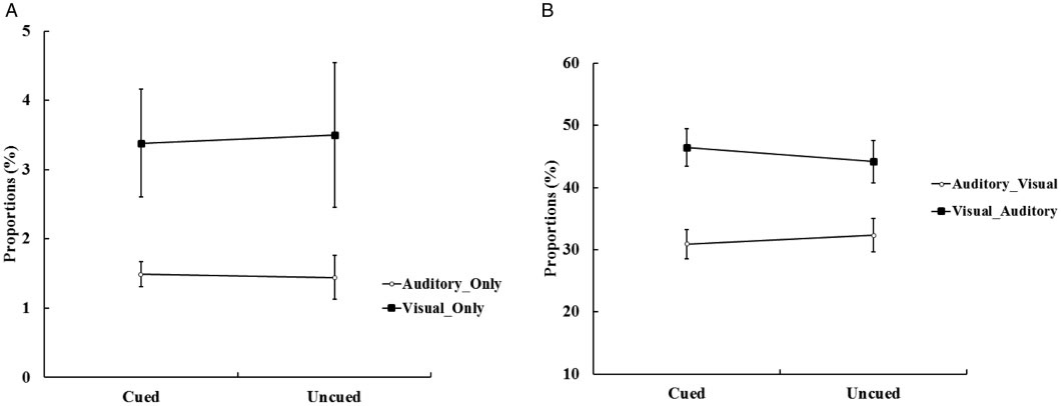
A: The proportions of the Visual_Only trials and the Auditory_Only trials at the cued and the uncued location. Visual_Only trials occurred more frequently than Auditory_Only. B: The proportions of the Visual_Auditory trials and the Auditory_Visual trials at the cued and the uncued location. The Visual_Auditory trials occurred more frequently than the Auditory_Visual trials at the cue location (the error bars indicate SEs).

The proportions of different types of bimodal trials in the present experiment were illustrated in Figure 2. First, we followed the first method of data analysis on the Colavita effect: The proportions of incorrect bimodal trials (i.e., Visual_Only and Auditory_Only bimodal trials) were submitted to a 2 (cue validity: cued vs. uncued) × 2 (type of incorrect bimodal trials: Visual_Only vs. Auditory_Only) repeated-measures ANOVA; this is shown in Figure 3A. The main effect of the type of incorrect bimodal trials was significant, *F*(1, 32) *=* 5.06, *p <* .05, $\eta_{\text{p}}^{2}$ *=* 0.14, indicating that the proportion of the Visual_Only trials (3%) was significantly larger than the Auditory_Only trials (2%), suggesting that there was the Colavita effect. The main effect of cue validity was not significant, *F* < 1. In addition, the interaction between the cue validity and the type of errors was not significant, *F* < 1. The size of the Colavita effect at the cued location (2%) and at the uncued location (2%) was not significant, *t <* 1.

Furthermore, for the correct and nonsimultaneous bimodal trials, we conducted the proportions of the Visual_Auditory and the Auditory_Visual trials to a 2 (cue validity: cued vs. uncued) × 2 (type of correctly responded bimodal trials: Visual_Auditory vs. Auditory_Visual) repeated-measures ANOVA; this is shown in Figure 3B. The main effect of the type of correctly responded bimodal trials was significant, *F*(1, 32) = 6.28, *p* < .05, $\eta_{\text{p}}^{2}$ = 0.16, indicating that the proportion of the Visual_Auditory trials (45%) was significantly higher than the proportion of the Auditory_Visual trials (32%), suggesting that there was the Colavita effect. The main effect of cue validity was not significant, *F* < 1. The interaction was significant, *F*(1, 32) = 4.59, *p* < .05, $\eta_{\text{p}}^{2}$ = 0.13. Paired *t* tests of simple effects revealed that the proportion of the Visual_Auditory trials (46%) was significantly higher than the proportion of the Auditory_Visual trials (31%) at the cued location, *t*(32) = 2.81, *p* < .05, Cohen’s *d* = 0.76. More importantly, the size of the Colavita effect was significantly larger at the cued location (16%) than the uncued location (12%), *t*(32) = 2.14, *p* < .05, Cohen’s *d* = 0.54. This indicated that endogenous spatial attention can regulate the size of the Colavita effect, and the size of the Colavita effect at the cued location was greater than the uncued location.

#### Reaction Time

**Figure 4.**
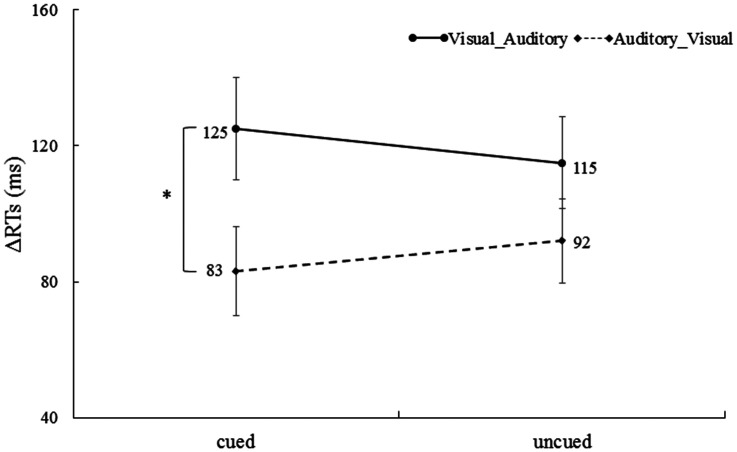
ΔRTs of the Visual_Auditory trials and the Auditory_Visual trials at the cued location and the uncued location. The ΔRT of the Visual_Auditory trials was significantly longer than ΔRT of the Auditory_Visual trials at the cue location (*p < .05. The error bars indicate SEs). RT = reaction time.

For RTs in the unimodal trials, a 2 (cue validity: cued vs. uncued) × (ΔRT of correctly responded bimodal trials: Visual_Auditory vs. Auditory_Visual) repeated-measures ANOVA was performed. The main effect of the cue validity was not significant, *F* < 1. The main effect of the ΔRT of correctly responded bimodal trials was significant, *F*(1, 32) = 5.05, *p* < .05, $\eta_{\text{p}}^{2}$ = 0.14, indicating that ΔRT of the Visual_Auditory trials (120 ms) was significantly higher than the ΔRT of the Auditory_Visual trials (87 ms), suggesting that there was the Colavita effect. The interaction was also significant, *F*(1, 32) = 4.98, *p* < .05, $\eta_{\text{p}}^{2}$ = 0.14. Further tests of simple effects showed that the ΔRT of the Visual_Auditory trials at the cued location (128 ms) was greater than the ΔRT of the Auditory_Visual trials at the cued location (83 ms), *t*(32) = 2.80, *p* < .05, Cohen’s *d* = 0.48 (Figure 4). More importantly, the size of the Colavita effect was significantly larger at the cued location (42 ms) than the uncued location (22 ms), *t*(32) = 2.23, *p* < .05, Cohen’s *d* = 0.23. This indicated that endogenous spatial attention can regulate the size of the Colavita effect, and the size of the Colavita effect at the cued location was greater than that at the uncued location.

By synthesizing the proportions and the RT results, we clearly found that the Colavita effect was stable at both cued and uncued locations. Specifically, regardless of whether we used the proportions of type of correctly responded bimodal trials or the RT to analyze the size of the Colavita effect, the size of Colavita effect was significantly larger at the cued location than the uncued location; this suggested that endogenous spatial attention enhanced the size of the Colavita effect.

## Experiment 2

In Experiment 1, the cue were arrows oriented to “left” or “right” in visual presentation, which can effectively regulate the direction of endogenous attention. However, due to the asymmetry between visual and auditory modalities, visual information and auditory information may be processed in different ways (Spence et al., 2000; Wang et al., 2020; Wu et al., 2019). Therefore, we conducted Experiment 2, changing visual cues to auditory cues, to further explore the influence of endogenous spatial attention on the Colavita effect.

### Method

#### Participants

We recruited 29 healthy volunteers (25 female, 4 male, aged 17–28 years) participated in the present experiment. They were all right handed, with normal hearing and normal or corrected-to-normal visual acuity. None of them had a history of neurological or psychiatric disorders. All participants gave informed consent prior to the experiment in accordance with the Helsinki declaration and were paid afterward. This experiment was approved by the Ethics Committee of Department of Psychology, Soochow University.

To test the suitability of the sample size, we performed a sensitivity analysis of the two-tailed paired sample *t* test in G*power 3.1.9.2 (Faul et al., 2007, 2009). Input parameters: ɑ err prob = 0.05, power (1–β err prob) = 0.80, and total sample size = 29.Output parameter: Cohen’s *d* = 0.54. The results showed that we have enough power to achieve moderate effect, so our sample size was appropriate.

#### Apparatus, Materials, and Procedure

The apparatus, materials, and procedure in Experiment 2 were exactly the same as those in Experiment 1 except that the cue was presented in auditory modal, rather than in visual modal. The cue was the sound of “left” or “right” by the voice that point to the left or right location; sound cues were presented in both ears through headphones.

### Results

#### Proportions of Different Types of Bimodal Trials

In bimodal trials in which participants made incorrect responses, we categorized two types of response according to the error type (“Visual_Only” or “Auditory_Only”). Since the error rates on the bimodal trials were too low, we did not analyze these effects with repeated-measures ANOVA with the factors of cue validity (cued vs. uncued) and error type (“Visual_Only” vs. “Auditory_Only”).

**Figure 5.**
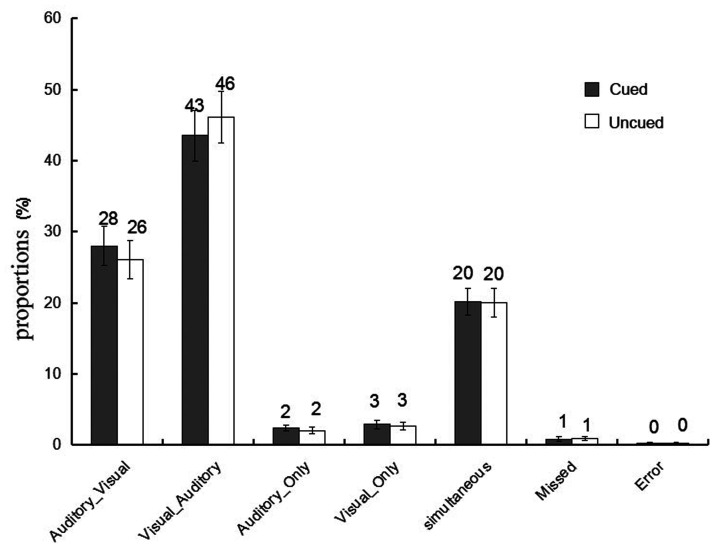
The proportions of the six different types of conditions in the bimodal trials at the cued and the uncued location. Visual_Auditory trials, in which visual responses preceded auditory responses; Auditory_Visual trials, in which auditory responses preceded visual responses; Visual_Only trials, in which only visual responses were made; Auditory_Only trials, in which only auditory responses were made; Simultaneous trials, in which participants responded simultaneously to the auditory and the visual components (RT difference less than 5 ms); and Missed trials, in which no responses were recorded (the error bars indicate SEs).

**Figure 6.**
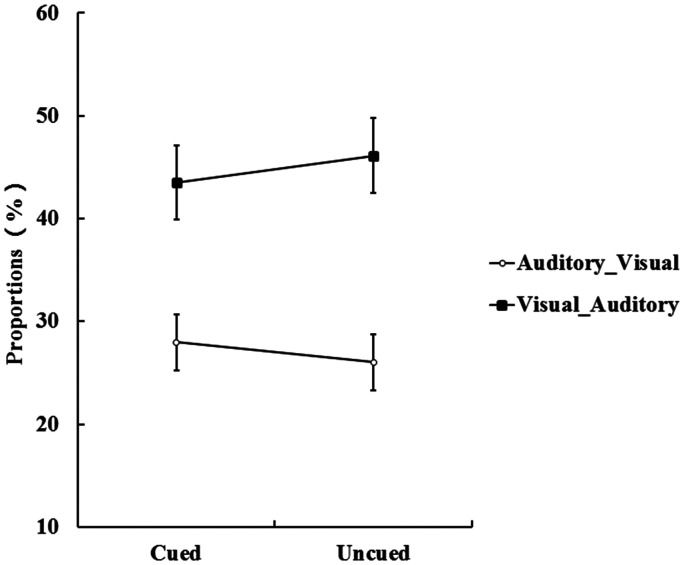
The proportions of the Visual_Auditory trials and the Auditory_Visual trials at the cued and the uncued location. The Visual_Auditory trials occurred more frequently than the Auditory_Visual trials at the uncued location (the error bars indicate SEs).

The proportions of different types of bimodal trials were illustrated in Figure 5. We conducted the proportions of the Visual_Auditory and the Auditory_Visual trials to a 2 (cue validity: cued vs. uncued) × 2 (type of correctly responded bimodal trials: Visual_Auditory vs. Auditory_Visual) repeated-measures ANOVA. The main effect of the type of correctly responded bimodal trials was significant, *F*(1, 28) = 8.90, *p* < .01, $\eta_{\text{p}}^{2}$ = 0.24, indicating that the proportion of the Visual_Auditory trials (45%) was significantly higher than the proportion of the Auditory_Visual trials (27%), suggesting that there was the Colavita effect. The main effect of cue validity was not significant, *F* < 1. Moreover, the interaction between cue validity and the type of correctly responded bimodal trials was significant, *F*(1, 28) = 5.63, *p* < .05, $\eta_{\text{p}}^{2}$ = 0.17 (Figure 6). Paired *t* tests of simple effects revealed that the proportion of the Visual_Auditory trials (46%) was significantly higher than the proportion of the Auditory_Visual trials (26%) at the uncued location, *t*(32) = 3.33, *p* < .05, Cohen’s *d* = 0.76. Moreover, the size of Colavita effect was larger at the uncued location (20%) than the cued location (16%), *t*(28) = 2.37, *p* < .05, Cohen’s *d =* 0.44. This indicated that endogenous spatial attention can regulate the size of the Colavita effect, and the size of the Colavita effect at the uncued location was greater than the cued location.

#### Reaction Time

**Figure 7.**
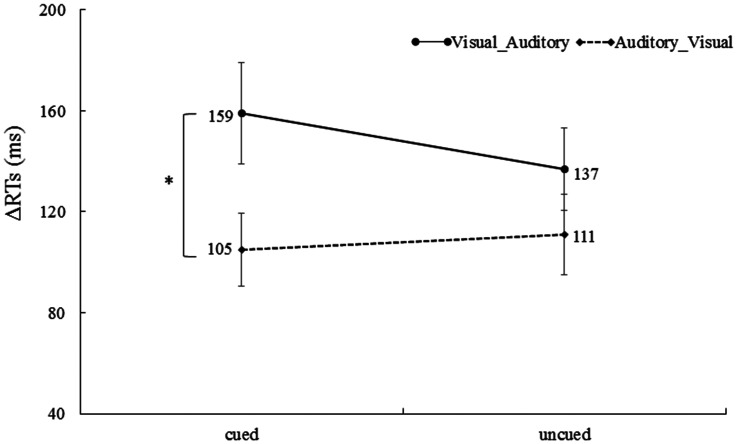
ΔRTs of the Visual_Auditory trials and the Auditory_Visual trials at the cued location and the uncued location. The ΔRT of the Visual_Auditory trials was significantly longer than ΔRT of the Auditory_Visual trials at the cue location (*p < .05. The error bars indicate SEs). RT = reaction time.

For RTs in the unimodal trials, a 2 (cue validity: cued vs. uncued) × (ΔRT of correctly responded bimodal trials: Visual_Auditory vs. Auditory_Visual) repeated-measures ANOVA was performed. The main effect of the cue validity was not significant, *F* < 1. The main effect of the ΔRT of correctly responded bimodal trials was significant, *F*(1, 32) = 4.77, *p* < .05, $\eta_{\text{p}}^{2}$ = 0.15, indicating that ΔRT of the Visual_Auditory trials (148 ms) was significantly higher than the ΔRT of the Auditory_Visual trials (108 ms), suggesting that there was the Colavita effect. The interaction was also significant, *F*(1, 32) = 6.90, *p* < .05, $\eta_{\text{p}}^{2}$ = 0.20. Further tests of simple effects showed that the ΔRT of the Visual_Auditory trials at the cued location (159 ms) was greater than the ΔRT of the Auditory_Visual trials at the cued location (105 ms), *t*(32) = 2.80, *p* < .05, Cohen’s *d* = 0.48 (Figure 7). More importantly, the size of the Colavita effect was significantly larger at the cued location (53 ms) than the uncued location (27 ms), *t*(28) = 2.63, *p* < .05, Cohen’s *d* = 0.49. This indicated that endogenous spatial attention can regulate the size of the Colavita effect, and the size of the Colavita effect at the cued location was greater than the uncued location.

To further investigate the effect of cue type (visual or auditory) on the proportions of response types of bimodal trials, we conducted a 2 (cue type: visual cues vs. auditory cues) × 2 (cue validity: cued vs. uncued) × 2 (type of correctly responded bimodal trials: Visual_Auditory vs. Auditory_Visual) repeated measure ANOVA. The results showed that the main effect of cue type was significant, *F*(1, 60) = 4.00, *p* = .05, $\eta_{\text{p}}^{2}$ = 0.06, and the proportion of visual cues (38%) was higher than that of auditory cues (36%); the main effect of response type was significant, *F*(1, 60) = 15.20, *p* < .001, $\eta_{\text{p}}^{2}$ = 0.20, and the proportion of the Visual_Auditory trials (45%) was higher than that of the Auditory_Visual trials (29%). The interaction of the three was significant, *F*(1, 60) = 10.29, *p* < .05, $\eta_{\text{p}}^{2}$ = 0.15. Other effects were not significant. More interestingly, we analyzed the “Simultaneous” trials for different types of cues by using independent sample *t* test; our results showed that *t*(60) = 3.01, *p* < .05, Cohen’s *d* = 0.54, and the proportion of auditory cues (20%) was higher than that of visual cues (15%). These results suggested that the cue type would affect the response of the participants. Compared with the visual cue, the participants made more simultaneous response under the auditory cue. These results suggested that visual and auditory cues were asymmetrical, although both two cue types could well guide endogenous spatial attention.

## Discussion

Our results showed that although we manipulated visual cues and auditory cues to guide endogenous spatial attention, the Colavita effect was always influenced by endogenous spatial attention, and the size of the Colavita effect was larger at the cued location than at the uncued location. We used three indexes to measure the size of the Colavita effect. These three indexes were derived from the following observations: (a) the proportion of Visual_Only trials was significantly higher than that of Auditory_Only trials (Colavita, 1974); (b) the proportion of Visual_Auditory trials was significantly higher than that of Auditory_Visual trials (Van Damme et al., 2009); and (c) the RT difference in Visual_Auditory trials was significantly greater than that in Auditory_Visual trials (Fang et al., 2020; Huang et al., 2015). In Experiment 1, we found that regardless of the index used, participants showed the classic Colavita effect. Perhaps due to too few trials involving the first index, we did not obtain significant results. However, using the second and third indexes, we found that the Colavita effect was produced at the cued location and that the size of the Colavita effect was larger at the cued location than at the uncued location. In Experiment 2, because the proportions of the Visual_Only trials and the Auditory_Only trials were too small, we did not analyze them; however, we used the second and third indexes and found that the Colavita effect was evident. However, we also obtained a seemingly paradoxical result: Employing the second index, we found that the Colavita effect was not produced at the cued location, and the size of the effect was larger at the uncued location than at the cued location, whereas with the third index, we found that the effect was produced at the cued location, and its size was larger at the cued location than at the uncued location.

In Experiment 2, we used the second and third indexes to measure the size of the Colavita effect and obtained seemingly opposite results. However, when we combined the results from Experiment 1 and Experiment 2, we found that the proportion of correctly responded bimodal trials in Experiment 1 was higher than that in Experiment 2, which meant that compared with those in Experiment 1, the participants in Experiment 2 had less Visual_Auditory trials and the Auditory_Visual trials. Moreover, we analyzed the proportion of simultaneous trials and found that the proportion in Experiment 2 was significantly higher than that in Experiment 1. As the participants in Experiment 2 had more simultaneous trials, they had fewer Visual_Auditory trials and Auditory_Visual trials, which affected the Colavita effect. The reason that the participants showed this response pattern under auditory cues may be that endogenous modality attention was involved in the multisensory integration task. Although auditory cues can direct attention to a specific spatial location, the auditory cues in this study had semantic meaning, which may have resulted in the participants’ partial attention remaining directed act the auditory modality for a short time. In addition, the Colavita effect is a visual dominant multisensory integration effect (Colavita, 1974; Sinnett et al., 2007). At the cued locations, attention to the auditory modality improved the participants’ ability to distinguish auditory stimuli, which led to the disappearance of the Colavita effect at these locations. This explanation is supported by previous studies (Sinnett et al., 2007; Vibell et al., 2007). Vibell et al. (2007) found that paying attention to a specific modality can accelerate information processing in low-level cortical areas; this effect is referred to as the prior-entry effect. Sinnett et al. (2007) varied the probability of the occurrence of targets in each sensory modality to assess its influence on the Colavita effect consequences. Their results demonstrated that selective attention to a particular sensory modality can modulate—although not completely reverse—visual dominance as illustrated by the Colavita effect.

Our results using the third index, the RT difference between the Visual_Auditory trials and the Auditory_Visual trials, showed that the size of the Colavita effect at the cued location was larger than that at the uncued location for both visual and auditory cues. This result is consistent with those of previous studies, suggesting that endogenous spatial attention can affect multisensory integration and that multisensory integration can be enhanced at the precued location (Fairhall & Macaluso, 2009; Senkowski et al., 2005; Talsma & Woldorff, 2005). The RT difference is essentially a manifestation of the psychological refractory period (Pashler, 1994a, 1994b); it refers to the fact that when individuals perform two tasks in succession, they tend to have a delayed response to the second task. Huang et al. (2015) found that the visual response recovered quickly in psychological refractory period caused by auditory response than vice versa. It can be used as a way to represent the visual dominance effect. Therefore, in our study, we used the RT difference as the third index of the Colavita effect. Compared with the third index, the first and second indexes demonstrated some shortcomings. Before the experiment began, the participants underwent a large number of practice trials, and in the formal experiment, they completed a large number of trials (40% visual stimuli, 40% auditory stimuli, and 20% bimodal stimuli). Therefore, in our experiment, visual-only trials and auditory-only trials accounted for a small proportion of the total trials, so the first index was not particularly appropriate. Furthermore, the second index may be influenced by the proportion of bimodal trials depending on whether visual or auditory cues are employed. Our results showed that the third index may be a reliable and effective index for measuring the size of the Colavita effect.

To summarize, this study adopted the cue-target paradigm to investigate the effect of endogenous spatial attention on the Colavita effect. The results showed that the size of the Colavita effect can be influenced by endogenous spatial attention: The size of the Colavita effect was greater when the participants’ endogenous attention was directed to the cued location than when it was directed to the uncued location under both visual cues and auditory cues. In addition, since the majority of the bimodal trials contained correct and nonsimultaneous responses, the visual dominance effect as defined in the present study provided a sufficient number of trials to investigate the potential neural mechanisms underlying the sensory dominance effect for future functional magnetic resonance imaging or event-related potential studies.

## References

[bibr1-20416695211027186] Alsius A. Navarra J. Campbell R. Soto-Faraco S. (2005). Audiovisual integration of speech falters under high attention demands. Current Biology, 15(9), 839–843. 10.1016/j.cub.2005.03.046 15886102

[bibr2-20416695211027186] Alsius A. Navarra J. Soto-Faraco S. (2007). Attention to touch weakens audiovisual speech integration. Experimental Brain Research, 183(3), 399–404. 10.1007/s00221-007-1110-1 17899043

[bibr3-20416695211027186] Colavita F. B. (1974). Human sensory dominance. Perception & Psychophysics, 16(2), 409–412. 10.3758/BF03203962

[bibr4-20416695211027186] Colavita F. B. Tomko R. Weisberg D. (1976). Visual prepotency and eye orientation. Bulletin of the Psychonomic Society, 8(1), 25–26. 10.3758/BF03337062

[bibr5-20416695211027186] Colavita F. B. Weisberg D. (1979). A further investigation of visual dominance. Attention, Perception, & Psychophysics, 25(4), 345–347. 10.3758/BF03198814 461094

[bibr6-20416695211027186] Coull J. T. Nobre A. C. (1998). Where and when to pay attention: The neural systems for directing attention to spatial locations and to time intervals as revealed by both PET and fMRI. Journal of Neuroscience, 18(18), 7426–7435. 9736662 10.1523/JNEUROSCI.18-18-07426.1998 PMC6793260

[bibr7-20416695211027186] de Gelder B. Bertelson P. (2004). Multisensory integration, perception and ecological validity. Trends in Cognitive Sciences, 8(1), 7. 10.1016/j.tics.2003.11.005 14550494

[bibr8-20416695211027186] Egeth H. E. Sager L. C. (1977). On the locus of visual dominance. Attention, Perception, & Psychophysics, 22(1), 77–86. 10.3758/BF03206083

[bibr9-20416695211027186] Fairhall S. L. Macaluso E. (2009). Spatial attention can modulate audiovisual integration at multiple cortical and subcortical sites. European Journal of Neuroscience, 29(6), 1247–1257. 10.1111/j.1460-9568.2009.06688.x 19302160

[bibr10-20416695211027186] Fang Y. Li Y. Xu X. T. Tao H. Chen Q. (2020). Top-down attention modulates the direction and magnitude of sensory dominance. Experimental Brain Research, 238(2), 587–600. 10.1007/s00221-020-05737-7 31996936

[bibr500-20416695211027186] Faul, F., Erdfelder, E., Buchner, A., & Lang, A.-G. (2009). Statistical power analyses using G*Power 3.1: Tests for correlation and regression analyses. *Behavior Research Methods, 41*(4), 1149–1160. https://doi.org/10.3758/BRM.41.4.1149 10.3758/BRM.41.4.1149 19897823

[bibr501-20416695211027186] Faul, F., Erdfelder, E., Lang, A.-G., & Buchner, A. (2007). G*Power 3: A flexible statistical power analysis program for the social, behavioral, and biomedical sciences. *Behavior Research Methods, 39*(2), 175–191. https://doi.org/10.3758/BF03193146 10.3758/bf03193146 17695343

[bibr11-20416695211027186] Giard M. Peronnet F. (1999). Auditory–visual integration during multimodal object recognition in humans: A behavioral and electrophysiological study. Journal of Cognitive Neuroscience, 11(5), 473–490. 10.1162/089892999563544 10511637

[bibr12-20416695211027186] Harrar V. Tammam J. Perez-Bellido A. Pitt A. Stein J. Spence C. (2014). Multisensory integration and attention in developmental dyslexia. Current Biology, 24(5), 531–535. 10.1016/j.cub.2014.01.029 24530067

[bibr13-20416695211027186] Hirst R. J. Cragg L. Allen H. A. (2018). Vision dominates audition in adults but not children: A meta-analysis of the Colavita effect. Neuroscience & Biobehavioral Reviews, 94, 286–301. 10.1016/j.neubiorev.2018.07.012 30048672

[bibr14-20416695211027186] Huang S. Li Y. Zhang W. Zhang B. Liu X. Mo L. Chen Q. (2015). Multisensory competition is modulated by sensory pathway interactions with fronto-sensorimotor and default-mode network regions. Journal of Neuroscience, 35(24), 9064–9977. 10.1523/JNEUROSCI.3760-14.2015 26085631 PMC6605163

[bibr15-20416695211027186] Kinchla R. A. (1974). Detecting target elements in multielement arrays: A confusability model. Perception & Psychophysics, 15(1), 149–158. 10.3758/BF03205843

[bibr16-20416695211027186] Koppen C. Alsius A. Spence C. (2008). Semantic congruency and the Colavita visual dominance effect. Experimental Brain Research, 184(4), 533–546. 10.1007/s00221-007-1120-z 17885751

[bibr18-20416695211027186] Koppen C. Spence C. (2007a). Spatial coincidence modulates the Colavita visual dominance effect. Neuroscience Letters, 417(2), 107–111. 10.1016/j.neulet.2006.10.069 17408855

[bibr19-20416695211027186] Koppen C. Spence C. (2007b). Audiovisual asynchrony modulates the Colavita visual dominance effect. Brain Research, 1186, 224–232. 10.1016/j.brainres.2007.09.076 18005944

[bibr20-20416695211027186] Koppen C. Spence C. (2007c). Seeing the light: Exploring the Colavita visual dominance effect. Experimental Brain Research, 180(4), 737–754. 10.1007/s00221-007-0894-3 17333012

[bibr21-20416695211027186] Koppen C. Spence C. (2007d). Assessing the role of stimulus probability on the Colavita visual dominance effect. Neuroscience Letters, 418(3), 266–271. 10.1016/j.neulet.2007.03.032 17398003

[bibr24-20416695211027186] Li C. Chen K. Han H. Chui D. Wu J. (2012). An fMRI study of the neural systems involved in visually cued auditory top-down spatial and temporal attention. PLoS One, 7(11), e49948. 10.1371/journal.pone.0049948 23166800 PMC3499497

[bibr25-20416695211027186] Li Q. Wu J. Touge T. (2010). Audiovisual interaction enhances auditory detection in late stage: An event-related potential study. NeuroReport, 21(3), 173–178. 10.1097/WNR.0b013e3283345f08 20065887

[bibr26-20416695211027186] Li Q. Yang H. Sun F. Wu J. (2015). Spatiotemporal relationships among audiovisual stimuli modulate auditory facilitation of visual target discrimination. Perception, 44(3), 232–242. 10.1068/p7846 26562250

[bibr27-20416695211027186] Li Y. Liu M. Zhang W. Huang S. Zhang B. Liu X. Chen Q. (2017). Neurophysiological correlates of visual dominance: A lateralized readiness potential investigation. Frontiers in Psychology, 8, 1–8. 10.3389/fpsyg.2017.00303 28303113 PMC5332361

[bibr28-20416695211027186] Mozolic J. L. Hugenschmidt C. E. Peiffer A. M. Laurienti P. J. (2008). Modality-specific selective attention attenuates multisensory integration. Experimental Brain Research, 184(1), 39–52. 10.1007/s00221-007-1080-3 17684735

[bibr29-20416695211027186] Pashler H. (1994a). Dual-task interference in simple tasks: Data and theory. Psychological Bulletin, 116(2), 220–244. 10.1037/0033-2909.116.2.220 7972591

[bibr30-20416695211027186] Pashler H. (1994b). Overlapping mental operations in serial performance with preview. Quarterly Journal of Experimental Psychology, 47(1), 161. 10.1080/14640749408401148 8177960

[bibr31-20416695211027186] Posner M. I. (1980). Orienting of attention. Quarterly Journal of Experimental Psychology, 32, 3–25. 10.1080/00335558008248231 7367577

[bibr34-20416695211027186] Schmid C. Buechel C. Rose M. (2011). The neural basis of visual dominance in the context of audio-visual object processing. NeuroImage, 55(1), 304–311. 10.1016/j.neuroimage.2010.11.051 21112404

[bibr35-20416695211027186] Senkowski D. Talsma D. Herrmann C. S. Woldorff M. G. (2005). Multisensory processing and oscillatory gamma responses: Effects of spatial selective attention. Experimental Brain Research, 166(3), 411–426. 10.1007/s00221-005-2381-z 16151775

[bibr36-20416695211027186] Sinnett S. Spence C. Soto-Faraco S. (2007). Visual dominance and attention: The Colavita effect revisited. Perception & Psychophysics, 69(5), 673–686. 10.3758/BF03193770 17929691

[bibr37-20416695211027186] Spence C. (2009). Explaining the Colavita visual dominance effect. Progress in Brain Research, 176, 245–258. 10.1016/S0079-6123(09)17615-X 19733761

[bibr39-20416695211027186] Spence C. Lloyd D. McGlone F. Nicholls M. E. R. Driver J. (2000). Inhibition of return is supramodal: A demonstration between all possible pairings of vision, touch, and audition. Experimental Brain Research, 134(1), 42–48. 10.1007/s002210000442 11026724

[bibr40-20416695211027186] Talsma D. (2015). Predictive coding and multisensory integration: An attentional account of the multisensory mind. Frontiers in Integrative Neuroscience, 9(19), 9. 10.3389/fnint.2015.00019 25859192 PMC4374459

[bibr41-20416695211027186] Talsma D. Doty T. J. Woldorff M. G. (2007). Selective attention and audiovisual integration: Is attending to both modalities a prerequisite for early integration? Cerebral Cortex, 17(3), 679–690. 10.1093/cercor/bhk016 16707740

[bibr42-20416695211027186] Talsma D. Senkowski D. Soto-Faraco S. Woldorff M. G. (2010). The multifaceted interplay between attention and multisensory integration. Trends in Cognitive Science, 14(9), 400–410. 10.1016/j.tics.2010.06.008 PMC3306770 20675182

[bibr43-20416695211027186] Talsma D. Woldorff M. G. (2005). Selective attention and multisensory integration: Multiple phases of effects on the evoked brain activity. Journal of Cognitive Neuroscience, 17(7), 1098–1114. 10.1162/0898929054475172 16102239

[bibr44-20416695211027186] Tang X. Li C. Li Q. Gao Y. Yang W. Yang J. Ishikawa S. Wu J. (2013). Modulation of auditory stimulus processing by visual spatial or temporal cue: An event-related potentials study. Neuroscience Letters, 553, 40–45. 10.1016/j.neulet.2013.07.022 23896527

[bibr45-20416695211027186] Tang X. Y. Wu J. L. Shen Y. (2016). The interactions of multisensory integration with endogenous and exogenous attention. Neuroscience & Biobehavioral Reviews, 61, 208–224. 10.1016/j.neubiorev.2015.11.002 26546734 PMC4753360

[bibr48-20416695211027186] Van Damme S. Crombez G. Spence C. (2009). Is visual dominance modulated by the threat value of visual and auditory stimuli? Experimental Brain Research, 193(2), 197–204. 10.1007/s00221-008-1608-1 18953530

[bibr50-20416695211027186] Vibell J. Klinge C. Zampini M. Spence C. Nobre A. C. (2007). Temporal order is coded temporally in the brain: Early event-related potential latency shifts underlying prior entry in a cross-modal temporal order judgment task. Journal of Cognitive Neuroscience, 19(1), 109–120. 10.1162/jocn.2007.19.1.109 17214568

[bibr51-20416695211027186] Wang A. Wu X. Tang X. Zhang M. (2020). How modality processing differences affect cross-modal nonspatial repetition inhibition. PsyCh Journal, 9(3), 306–315. 10.1002/pchj.332 31908147

[bibr53-20416695211027186] Wu J. Li Q. Bai O. Touge T. (2009). Multisensory interactions elicited by audiovisual stimuli presented peripherally in a visual attention task: A behavioral and event-related potential study in humans. Journal of Clinical Neurophysiology, 26(6), 407–413. 10.1097/WNP.0b013e3181c298b1 19952565

[bibr54-20416695211027186] Wu J. Yang J. Yu Y. Li Q. Nakamura N. Shen Y. Ohta Y. Yu S. Abe K. (2012). Delayed audiovisual integration of patients with mild cognitive impairment and Alzheimer’s disease compared with normal aged controls. Journal of Alzheimer’s Disease, 32(2), 317–328. 10.3233/JAD-2012-111070 PMC3746512 22810093

[bibr55-20416695211027186] Wu X. Wang A. Tang X. Zhang M. (2019). Different visual and auditory latencies affect cross-modal non-spatial repetition inhibition. Acta Psychologica, 200, 102940. 10.1016/j.actpsy.2019.102940 31665621

[bibr57-20416695211027186] Yue Z. Jiang Y. Li Y. Wang P. Chen Q. (2015). Enhanced visual dominance in far space. Experimental Brain Research, 233(10), 2833–2843. 10.1007/s00221-015-4353-2 26080757

